# Immunization against *Leishmania major *infection in BALB/c mice using a subunit-based DNA vaccine derived from TSA, LmSTI1, KMP11, and LACK predominant antigens

**DOI:** 10.22038/IJBMS.2019.14051

**Published:** 2019-12

**Authors:** Ghodratollah Salehi-Sangani, Mehdi Mohebali, Vahid Jajarmi, Ali Khamesipour, Mojgan Bandehpour, Mahmoud Mahmoudi, Hadi Zahedi-Zavaram

**Affiliations:** 1 Department of Medical Parasitology and Mycology, School of Public Health, Tehran University of Medical Sciences, Tehran, Iran; 2 Department of Medical Parasitology and Mycology, Faculty of Medicine, Mashhad University of Medical Sciences, Mashhad, Iran; 3 Center for Research of Endemic Parasites of Iran (CREPI), Tehran University of Medical Sciences, Tehran, Iran; 4 Department of Medical Biotechnology, School of Advanced Technologies in Medicine, Shahid Beheshti University of Medical Sciences, Tehran, Iran; 5 Cellular and Molecular Biology Research Center, Shahid Beheshti University of Medical Sciences, Tehran, Iran; 6 Centre for Research and Training in Skin Diseases and Leprosy, Tehran University of Medical Sciences, Tehran, Iran; 7 Department of Biotechnology, School of Medicine, Shahid Beheshti University of Medical Sciences, Tehran, Iran; 8 Department of Epidemiology and Biostatistics, School of Public Health, Tehran University of Medical Sciences, Tehran, Iran; 9 Department of Medical Parasitology and Mycology, School of Medicine, Shahid Beheshti University of Medical Sciences, Tehran, Iran

**Keywords:** BALB/c mice, Cytokines, DNA, Epitopes, Leishmania major, Vaccines

## Abstract

**Objective(s):**

To design a multivalent DNA vaccine encoding the most immunogenic regions of the *Leishmania major* antigens including TSA (Thiol-specific antioxidant protein), LmSTI1 (*Leishmania major* stress-inducible protein1), LACK (*Leishmania* homologue of receptors for activated C Kinase), and KMP11 (kinetoplastid membrane protein-11) on BALB/c mice.

**Materials and Methods:**

The chimeric construct was generated including the most immunogenic epitopes containing a combination of domains and oligopeptides of the aforementioned genes. The construct was cloned into pcDNA 3.1 plasmid and named “pleish-dom.” Following intramuscular injection of mice, the capability of the vector pleish-dom alone and with pIL-12 (expressing murine IL-12) to raise protective cytokines and parasite burden was evaluated in the BALB/c mice as a susceptible animal model against *L. major*.

**Results:**

The immunized mice with pleish-dom/pIL-12 showed the highest and the lowest levels of interferon-gamma (IFN-γ) and interleukin-10 (IL-10), as well as the lowest leishmanin skin test (LST) reactions, were found through 8 weeks post-infection.

**Conclusion:**

Although the obtained DNA vaccine from the immunogenic chimeric protein of *L. major* antigens was able to induce a high level of IFN-γ, it partially protected mice against L. major. However co-administration with pIL-12 led to shift immune response to Th1 phenotype, granuloma formation, and lowered parasite burden, and consequently distinct protection was found.

## Introduction

Nowadays, it is estimated that a 10^th^ of the world population is at risk of leishmaniasis infection, 12 million people are infected, and 2 million new cases annually are reported. Leishmaniasis is an increasing public health hazard in many countries around the world (1). Up to now, treatment of leishmaniasis has been the main challenge of control strategy. Although other programs such as personal protection and health education, vectors, and reservoirs control have led to reduced incidence of leishmaniasis in endemic areas, finding an effective and definite vaccine has been unsuccessful (2). Various methods such as live and whole killed parasites (3-5), parasite fractions(6, 7), live attenuated parasites (8, 9), recombinant proteins (10, 11) and DNA vaccines (12) have been used as vaccine with limited success.

DNA vaccines are classified as the third generation vaccines, which are interesting due to the capability to induce cellular and humoral immune responses. This approach has been applied for the prophylaxis of several infectious and non-infectious diseases in the last decade (13). In spite of efforts, no human DNA vaccine has been approved yet; several recombinant proteins have been introduced for veterinary applications including Leishmune (14), Leish-Tec (15), and CaniLeish (16). Designing a DNA vaccine for a parasitic disease is more complicated than bacterial or viral ones due to the complexity of the life cycles, antigenic variations, stage-specific immunity, and other immune evasion mechanisms existing in parasites (17).

Over the last two decades, numerous *Leishmania* candidate vaccines have been introduced (18) and many different *Leishmania* antigens including gp63, LACK, TSA, CPa, CPb, TRYP, NH36, LmSTI1, KMP11, PFR-2, LeIF, histones, and others have been evaluated as vaccine candidates with variable levels of protective immune responses in a variety of experimental models of leishmaniasis (19-21). Many reports suggested that immunization with a combination of several antigens may extend the immunogenicity of a vaccine and improve protective immune responses (20, 22). To this end, either a plasmid consisting of coding sequence of several genes or two or more plasmids with genes of interest can be co-administered. Leish-111f, Leish-110f, Q-Protein, and KSAC are cocktail vaccines composed of several distinct antigens of *Leishmania* that have been evaluated in animal models, and Leish-111f has reached phase I in human clinical trials (23). But a more advantageous strategy to exploitation of the maximum immunogenicity of several antigens is identification and utilization of major histocompatibility complex (MHC)-binding epitopes or peptides. Antigens usually include several immunodominant epitopes that induce immune response system through the presentation on the antigen-presenting cells (APCs) surface by the MHC molecules. Recognition of these epitopes could develop epitope-based vaccines that improve efficiency and effectiveness of immune system responses (24).

Also, some strategies might be employed to improve efficacy of DNA vaccines against parasitic diseases such as use of genetic adjuvants, multivalent vaccines, efficient promoters, codon optimization, and prime–boost strategies(25, 26). It is shown that IL-12 is an immunomodulatory potent cytokine that plays a crucial role in the initiation and maintenance of Th1 responses via induction of IFN-γ production by T and NK cells. The effect of IL-12 is increased when administrated in combination with MHC-binding peptides, which leads to an antigen-specific Th1 recall response (27). 

In the current study, a multivalent DNA vaccine including immunogenic domains/oligopeptides of four prominent leishmanial genes; TSA, LmSTI1, KMP11, and LACK was designed and its ability to induce immune response and protection against *Leishmania major *infection with or without IL-12 expressing plasmid evaluated in a murine model of BALB/c mice.

## Materials and Methods

***Construct design and cloning***

**Figure 1:**
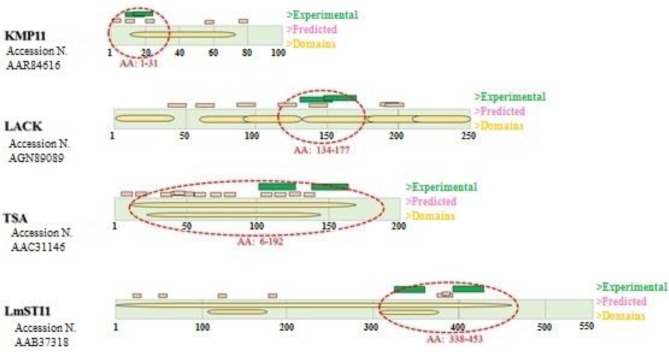
Antigen structures with the number of protein sequence are shown in rectangles and domains within it. Experimental and predicted epitopes have been displayed in green and pink color, respectively above the rectangle. Selected Domains/oligopeptides in any antigen have been marked with red dash lines

**Figure 2:**
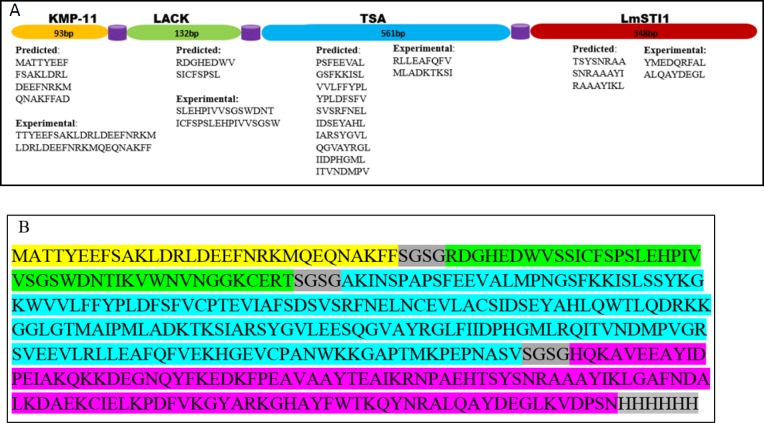
A schematic representation of the construct. A. Selected fragments of each protein with the number of nucleotides have been shown as linear successive domains separated by flexible linkers. The predicted and experimental validated peptides have been listed under each fragment. B. The sequence of the chimeric construct with linkers and His-tag

To obtain an efficient construct, the sequences of the mentioned genes; KMP11 (GeneBank accession number AY490814), LACK (GenBank accession number KC869693, TSA (GenBank accession number AF044679), and LmSTI1 (GenBank accession number U73845) were collected from NCBI. Then the predicted MHC class I binding epitopes with a higher score were selected from the databank (www.iedb.org). Moreover, the regions of the genes that were highly immunogenic were chosen through a search of the literature (Figure 1) (19, 20, 22, 28-32). Then the domain or domains containing the most antigenic epitopes were selected and assembled through flexible linkers (Figure 2A). Additionally, for expression analysis of the chimeric gene, a sequence encoding His-tag was added at the end of the construct (Figure 2B). The obtained sequence was synthesized by Shine Gene BioTechnologies Company (Shanghai, China) and sub-cloned into eukaryotic expression plasmids including pcDNA 3.1 and pEGFP-N1 as vectors for *in vivo* immunization and *in vitro* evaluation of the expression, respectively. To obtain fusion of the chimeric protein and GFP, the stop codon of the end of the construct was removed. pcDNA plasmid containing the construct was called pleish-dom.

***Subcloning and plasmid purification***

Synthesized plasmid (pGH) was transformed into *Escherichia coli,* top10 competent cells and subsequently was extracted from the bacterial pellet using a NucleoBond endotoxin-free plasmid DNA extraction kit (Macherey-Nagel, Düren, Germany). The plasmid was digested using *BamHI* restriction enzyme  (Jena Bioscience, Germany), and the resulting fragment of DNA was extracted from the gel using NucleoSpin, PCR clean-up, gel extraction kit (Macherey-Nagel, Duren, Germany) and subcloned into the *BamHI* restriction site of pcDNA3.1 and pEGFPN1 (Invitrogen) downstream of the CMV promoter. Plasmid encoding murine IL-12 (pIL-12) was kindly gifted by Dr. Azizi (Zabol University of Medical Sciences)(33). 

In order to prepare the required DNA vaccine for *in vivo* studies, plasmid extraction of pleish-dom was carried out on a large scale with NucleoBond Xtra Maxi EF (Macherey–Nagel, Düren, Germany).

***In vitro*** **expression**

To confirm the expression of construct in a mammalian cell line, pEGFP-N1 containing the construct (pEGFP-leish-dom) was linearized using *EcoO109I* restriction enzyme (New England BioLabs) and transfected into HEK293 cell line using lipofectamine 2000 (Invitrogen), according to the manufacturer’s instructions with minor modifications. Briefly, 2×10^5^ cells were cultured (500 µl per well) in a 24-well plate to achieve 60–65% confluency on the day of transfection. Then, the media was removed and replaced with a fresh serum-free DMEM without antibiotics, 2 hr before transfection. A mixture of lipofectamine 2,000 and pEGFP-leish-dom with a ratio of 3:1 was added into the cells. The transfected cells were incubated overnight at 37 ^°^C with 5% CO_2_. After 14–15 hr, the medium was replaced with fresh medium. GFP expression was monitored 24–48 hr post-transfection under a fluorescence microscope. 

***Western blot analysis***

Following preparation of the pleish-dom plasmid and before proceeding to the immunization step, the capability of the plasmid to express the desired protein was confirmed using Western blot analysis. Primarily, in order to generate a stable expressing cell line, *ScaI* enzyme (Jena Bioscience, Germany) was used for linearization of the pleish-dom plasmid. Transfected cells were treated with G418 (Gibco, Invitrogen) up to 600 µg ml^-1^ for two weeks. Then, the cells were trypsinized, collected and centrifuged for 10 min at 2500 g. The cell lysate was prepared using lysis buffer followed by sonication on ice. The separated protein bands using SDS-PAGE were transferred into the nitrocellulose membrane. Then, Western blot analysis was performed using an anti-His-tag mouse monoclonal antibody (Abcam) as described previously (34). 

***Animals and parasites***

Animal experiments were approved by the Ethical Committee of Tehran University of Medical Sciences (ID no. 240/1014). Female 6 to 8 week old BALB/c mice were purchased from Razi Vaccine and Serum Research Institute (Karaj, Iran). The animals were maintained in standard conventional conditions.

The *L. major *(MRHO/IR/75/ER) was used in this study. The virulence of *Leishmania *parasites was maintained by passage in BALB/c mice. *L. major *amastigotes were collected from the spleen of the infected mice and cultured in NNN (Novy-MacNeal-Nicolle) medium, incubated at 25+1 ^°^C and then subcultured in RPMI 1640 medium (Gibco, NY, U.S.A.) supplemented with 10 % fetal calf serum (Gibco), 100 U/ml of penicillin G, and 100 μg ml^-1^ of streptomycin sulphate (Gibco). Promastigotes were harvested at stationary phase, and 1x10^6 ^promastigotes in 50 μl were inoculated into the base of the tail with 1×10^6 ^*L. major* in 100 μl.

***Immunization and challenge of mice***

BALB/c mice were divided into four groups (9 mice per group), one group was injected with 100 µg pleish-dom alone, one group was injected with pleish-dom combined with plasmid encoding IL-12 (50 µg+50 µg, pIL-12), another group received PBS, and one group received 100 µg empty pcDNA intramuscularly in the hind leg. The booster injections were given three times in 2-week intervals. Four weeks after the last booster injection, different groups of mice were challenged subcutaneously at the base of the tail with 1×10^6 ^*L. major *promastigotes harvested at stationary phase. Lesion development was monitored, and the size of the lesion was recorded weekly using a Vernier caliper.

***Parasite burden***

In order to determine the parasite load, the serial dilution assay was performed for assessment of spleen parasite burden as described previously (35, 36). In brief, a piece of spleen about 5–10 mg was excised, weighed, and homogenized in RPMI medium supplemented with 20% heat-inactivated FCS, 100 U ml^-1^ penicillin, and 100 μg ml^-^1 streptomycin. Each tissue sample was diluted in a five-fold series in a 96-well plate. After ten days of incubation at 26 ^°^C, the plates were examined for the presence or absence of promastigotes using an inverted microscope. The parasite burden per organ was calculated as follows: (the reciprocal of the highest dilution at which promastigotes were detected/homogenized tissue weight) × organ weight.

***Cytokines evaluation***

Three mice from each group before and at week 8 after the challenge were sacrificed. The spleen from each mouse was aseptically removed and homogenized. The red blood cells were lysed using ACK lysis buffer (0.15 M NH4Cl, 1.0 mM KHCO3, 0.1 mM Na2EDTA, pH 7.4) and the remaining cells were washed with PBS buffer. Mononuclear cell suspension was cultured in DMEM+10% FCS and stimulated with Soluble *Leishmania* Antigen (SLA) (25 μg ml^-1^). The supernatants were collected at 48 and 72 hr for IL-10 and IFN-γ titration, respectively. The levels of cytokines were assessed by enzyme-linked immunosorbent assay (ELISA) using a commercial kit (R&D System Elisa kits) with protocols provided by the manufacturer.

**Figure 3:**
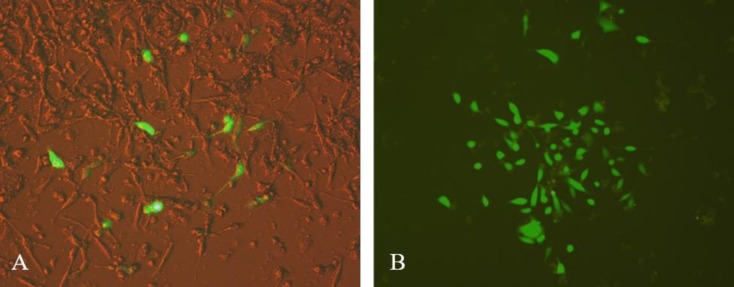
GFP expression of pEGFP-leish-dom in HEK293 cells. A: A merged picture of HEK293 cells after 48 hr transfection; B: G418-treated cells after two weeks incubation with an expanding colony

**Figure 4:**
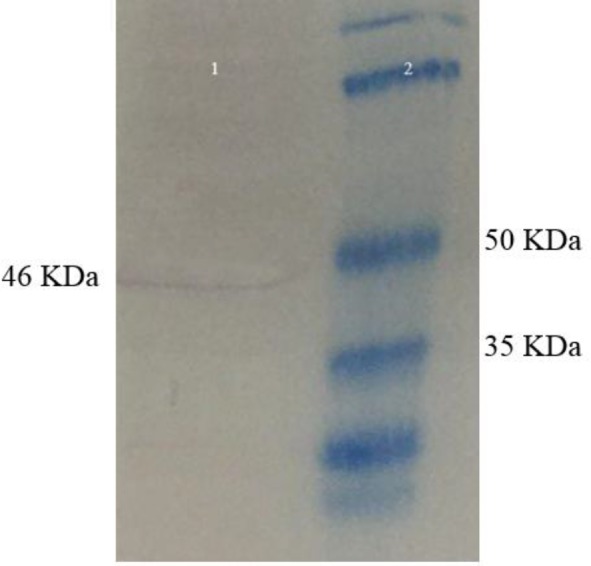
Western blotting analysis. Lane 1: The stable transfected Hek293 cells by pleish-dom with the desired protein band (46 kDa); Lane 2: protein marker

**Figure 5:**
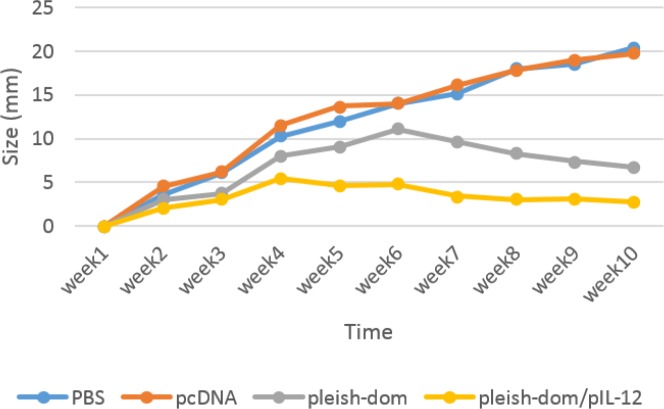
Lesion sizes were measured in experimental groups of animals for ten weeks post-infection. Lesion development was ceased at weeks 4 and 6 post-infection in pleish-dom/pIL-12 and pleish-dom groups, respectively. There was a significant difference (*P*<0.05)between the pleish-dom/pIL-12 group and control groups

**Figure 6:**
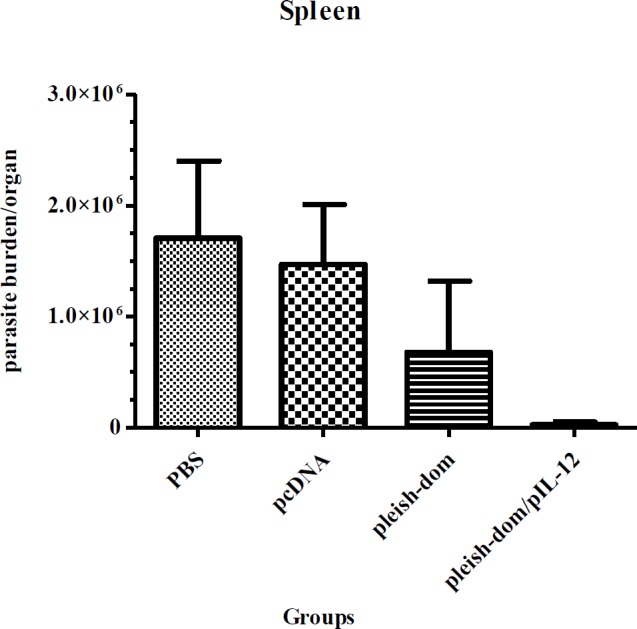
Spleen parasite burden in experimental groups. Mice were inoculated 3 times with pleish-dom alone and with pIL-12 as vaccinated groups and with pCDNA plasmid and PBS as control groups with a 2-week interval. Mice were challenged with 106 metacyclic promastigotes 4 weeks post last time of immunization. Spleen parasite burdens were evaluated 8 weeks post-challenge. Vaccination with pleish-dom/pIL-12 was able to prevent visceralization of the parasites in almost all the immunized mice except one mouse with a little parasite burden

**Figure 7:**
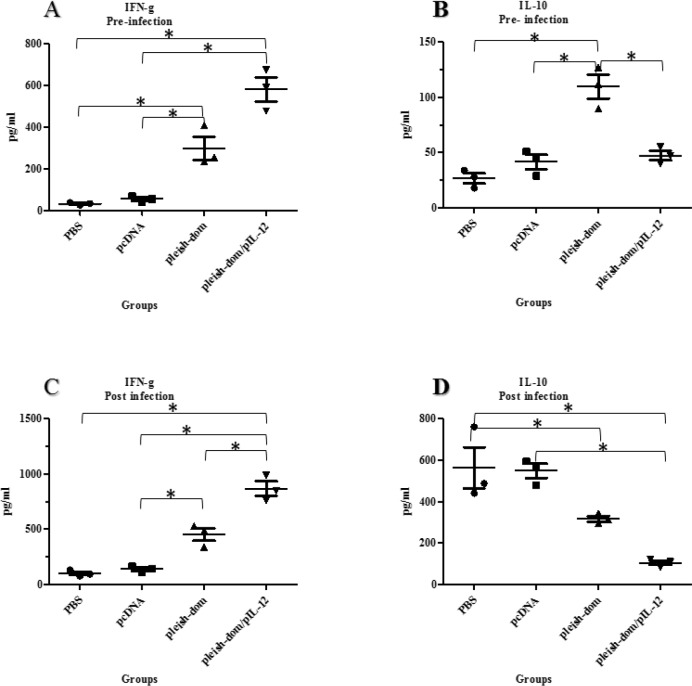
Cytokine assessment in the supernatant of spleen cell cultures. Extracted spleen cells were stimulated with soluble Leishmania antigen (25 μg/mL) for 48 hr and 72 hr for IL-10 and IFN-γ, respectively. The levels of cytokines were determined using ELISA. Tukey’s test followed by one way ANOVA was used to perform statistical analysis (* *P*≤0.05); A. increased level of the IFN-γ level in vaccinated groups before challenge; B. Higher level of IL-10 in mice vaccinated with pleish-dom alone before challenge; C. 4-fold increasing of IFN-γ level in mice vaccinated with pleish-dom/pIL-12 than before challenge; D. elevated level of IL-10 in control groups at 8 weeks post-challenge compared to before challenge

**Figure 8:**
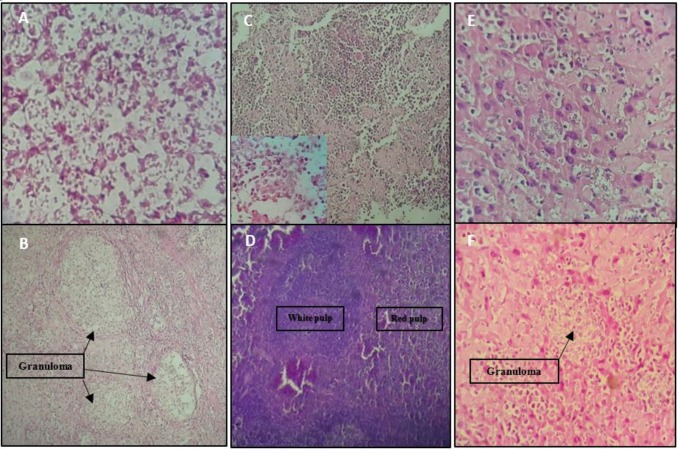
Microscopic examination: Site of infection; diffuse macrophages with plenty of internalized amastigotes infiltrated with a few polymorphs and lymphocytes without or rare granuloma formation in the site of infection of control groups(A) and many granulomas with entrapped amastigotes in vaccinated groups (B). Spleen; visceral *Leishmania* infection in control groups’ mice altered the histological structure of the spleen, led to the loss of cell populations in the spleen, and disorganized architecture with indistinct border between red and white pulps (C) versus regular architecture in spleen of mice vaccinated with pleish-dom/pIL-12(D). Liver; immature granuloma formation with diffused amastigotes out of granuloma in liver sections of control groups (E) and the parasitized multinucleated granulomas seen in the liver sections of mice vaccinated with pleish-dom (F)

***Histopathological examination***

To study the pathology response of challenged mice, a portion of the livers and spleens of a different group of mice were removed at week 8 post-challenge. The samples were fixed in 10% buffered formalin, embedded in paraffin and sliced in 5-μm thick sections mounted on microscope slides. The sections were stained with hematoxylin and eosin (H&E) and examined using light microscopy. 

***Statistical analysis***

One-way ANOVA analysis (Multiple comparisons Tukey’s *post hoc* test) was performed using the GraphPad Prism software. A value of *P*<0.05 was considered to be significant.

## Results

***Epitope identification***

Predicted MHC class-I binding epitopes were defined based on the sequences of antigens. A chimeric protein of successive domains of the candidate genes was designed as depicted in Figure 2. 

***Cloning and expression***

Both pleish-dom and pEGFP-leish-dom plasmids were successfully prepared by cloning the construct in pcDNA3.1 and pEGFPN-1 plasmids, respectively and verified by sequencing. Following transfection of the HEK293 cell line with pEGFP-leish-dom plasmid, cells were incubated for 24 hr at 37^ °^C and 5% CO_2_ and then monitored for expression of GFP under a fluorescent microscopy (Figure 3A). Colonies of positive G418-treated cells were observed 2 weeks post-transfection, which validates the stable transfection (Figure 3B). The data confirmed that the appropriate amount of expression of the chimeric protein existed.

To evaluate the capability of the expression system, cell lines were transfected and treated with G418 antibiotic for two weeks. As shown in Figure 4, Western blot analysis of the cell lysate confirmed an immunoreactive band of 46 kDa corresponding to the size of the chimeric protein.

***Lesion development***

The development of lesions in all groups of mice was monitored for eight weeks post-challenge. The results showed that lesion development was hampered in the group of mice immunized with pleish-dom/pIL-12 compared to the control groups. Statistically, a significant difference (*P*<0.05) was seen between the pleish-dom/pIL-12 group and the control groups (Figure 5). 

***Spleen parasite burden***

The parasite burden in the spleen of different groups of mice was evaluated at week eight post-challenge. The results showed that the group of mice in the control groups had a significantly higher parasite burden in the spleen, whereas the group of mice immunized with pleish-dom/pIL-12 showed a significantly lower parasite burden (*P*<0.05). Also mice immunized with pleish-dom alone did not exhibit a remarkably lower parasite burden (Figure 6) (*P*=0.168). 

***Cytokine results***

To determine the type of immune response induced by immunized animals, the levels of IFN-γ and Il-10 were measured as indicators of Th1 and Th2 immune response, respectively. Production of IFN-γ and IL-10 was assessed at two time points; at week four after last booster injection before challenge and at eight weeks after challenge. The results of the cytokine assays before challenge (Figure 7A) showed that there is a significant difference between the mean of IFN-γ in control groups that received PBS or empty pCDNA and the groups of mice vaccinated with pleish-dom alone or pleish-dom plus pIL-12 (*P*<0.01). The mean IL-10 (Figure 7B) before challenge was significantly (*P*<0.05) higher in the group of mice immunized with pleish-dom alone or with IL-12 compared with other groups. Increased level of IFN-γ was detected at eight weeks after challenge in all groups of mice. But the level of IFN-γ was significantly higher in the group of mice vaccinated with pleish-dom alone or with IL-12 (*P*<0.05) (Figure 7C). Also, the least increase of IL-10 level was attributed to mice immunized with pleish-dom plus pIL-12. Not only there was a significant difference (*P*<0.01) in the IL-10 level between vaccinated and control groups, but also a remarkable difference (*P*<0.01) was observed between both vaccinated groups at eight weeks post-challenge (Figure 7D). The ratio of IFN-γ/IL-10 was 0.2 for control groups against vaccinated groups with pleish-dom alone or with IL-12 were 1.4 and 8.1, respectively at eight weeks post-challenge. The results validated an IFN-γ-dominated Th1 response in mice immunized with pleish-dom plus pIL-12 against a dominant Th2 response in the control group.

***Microscopic histopathological findings***

In the prepared sections from tissues of infection sites of the control mice group, an acute inﬂammatory response with no granuloma formation was observed. Most of the cells seen in the inﬂammatory inﬁltrate of the mice of control groups were diffused macrophages that showed large parasitophorous vacuoles containing many amastigote parasites (Figure 8A). Mice vaccinated with pleish-dom presented a mixed inﬂammatory response composed of many macrophages and lymphocytes forming granulomas. The highest number of granulomas was seen in the infection site of mice vaccinated with pleish-dom/pIL-12 (Figure 8B). Also, many small lymphocytes, plasma cells, and granulocytes were seen with less parasitized macrophages. Microscopic histopathologic examination showed significant alterations in various groups of mice. 

In the stained liver sections control groups’ mice were observed a few granulomas consisting of a central macrophage core containing plenty of amastigotes inside and outside granulomas. Fibroblasts and mononuclear cells were present at the periphery of the granulomas (Figure 8E). Mature granulomas with different sizes were seen in the liver of the vaccinated with pleish-dom group mice (Figure 8F). Microscopic examination of the liver sections of the mice vaccinated with pleish-dom/pIL-12 showed normal morphology free of any inflammatory foci or parasites.

Microscopic examination of the spleen sections of the control group showed extensive disorganization of splenic tissue with infiltration of macrophages containing amastigotes so that there was no distinct border between white and red pulps (Figure 8C).The group of mice immunized with pleish-dom presented a partial disruption in the cellular architecture of the spleen. Infected macrophages were seen. Splenic architecture in the group of mice receiving pleish-dom/pIL-12 showed normal morphology and was free of parasites (Figure 8D).

## Discussion

Leishmaniasis remains a significant public health problem in some of the endemic foci(37, 38). Due to ongoing emergence of new foci, massive burden at the individual and community levels, and increasing drug resistance, alternative therapeutic strategies such as vaccines may help to alleviate these complications. To date, despite plenty of efforts by vaccine researchers no vaccine has been introduced against leishmaniasis (39). During the past years, different strategies, such as subunit-based vaccines, have been applied for immunization against varied species of *Leishmania* (40). It is possible to employ a broader range of protective epitopes as a chimeric protein by utilizing different *Leishmania* antigens (41). Maspi *et al*. evaluated capability of DNA vaccines containing LeIF, LACK, and TSA genes as a cocktail, fusion, and each gene alone to induce a Th1 immune response and suggested that vaccinated mice with cocktail and fusion forms represented a high level of IFN-γ and IgG2a as well as decreased mean size of lesions compared with genes alone form (42). In this study a stronger Th1 immune response against *L. major* was induced in mice vaccinated with LACK and TSA (genes used in our study) compared with the LeIF group. Ahmed *et al*. reported full protection against a low dose of  *L. major* in BALB/c mice vaccinated with a cocktail DNA vaccine composed of four *leishmania* antigens; LACKp24, TSA, LmSTI1, and CPa (12). Campos-Neto *et al*. showed that mice vaccinated with TSA and LmSTI1 genes either as a single DNA vaccine or a fusion construct were protected against *L. major*(20). Researchers generated Leish-111f, a recombinant polyprotein vaccine, that induced full protection against cutaneous and visceral leishmaniasis in murine and nonhuman primate models (10, 43). 

 Identification of the potential inducer for Th1 immune response has a crucial role in designing vaccines against *Leishmania* infection (23). This strategy may be a promising approach to produce an effective and multivalent vaccine. In the current work several different *Leishmania* antigens were considered as candidates whose potency had already been validated experimentally *in vivo* or via *in silico* analysis. We designed a chimeric protein composed of immunogenic domains/oligopeptides of four *Leishmania* antigens, including TSA, LmSTI1, LACK, and KMP11 that are present in both amastigote and promastigote forms. TSA was the only gene the sequence of which was selected almost entirely because of a lot of predicted epitopes as shown in Figure 2. According to several publications, these are the most promising antigens among vaccine candidates against *Leishmania spp* because of their potentials to be more protective and immunogenicity (19, 20, 44). 

The current study was conducted to evaluate the IFN-γ and IL-10 levels as characteristic indicators of Th1 and Th2 cells to determine the association of the immune response phenotype with the level of protection in different groups of mice. Our designed DNA vaccine was able to cause partial protection against cutaneous leishmaniasis *in vivo*. However, co-administration with plasmid expressing IL-12 resulted in a significant Th1-immune response. As a rule, the induced immune response phenotype determines the level of protection against cutaneous leishmaniasis(45). Induction of a strong Th1-immune response is necessary for complete protection against *L. major* that is yielded with the presence of IFN-γ in the absence of IL-10(46) as the model seen in the mice of pleish-dom/pIL-12 group. In contrast, a low ratio of IFN-γ/IL-10 causes the immune response shift towards Th2 immune phenotype in control groups. 

Data showed that superior protection against *L. major* acquired through strong Th1 immune response correlated with a vigorous IFN-γ level versus negligible level of IL-10 in mice vaccinated with pleish-dom/pIL-12. Also partial protection was provided in mice vaccinated with pleish-dom. Although the level of IFN-γ was elevated in this group, the ratio of IFN-γ/IL-10 was lower compared with pleish-dom/pIL-12 group. The ratio of IFN-γ/IL-10 in control groups was quite opposite the vaccinated groups. The mice of control groups exhibited a remarkable Th2 immune response as a consequence of a high level of IL-10 and insignificant level of IFN-γ. Therefore, a predominant Th1, mixed Th1/Th2, and polarized Th2 immune responses were induced in mice vaccinated with pleish-dom/pIL-12 pleish-dom and control groups, respectively. These results show a correlation between protection and the cytokine proﬁle in the groups under study.

 IL-12 has a pleiotropic effect on natural killer (NK) and T cells such as induction of proliferation, cytokines secretion and cytotoxic activity (47). The fundamental role of IL-12 has been demonstrated in the promotion of a Th1 response, and it has been administrated as an effective adjuvant in vaccine studies (48). Differentiation of Th0 phenotype into Th1 is mediated by IL-12, and as a result a Th1 immune response mounts through increasing IFN-γ production by NK and T cells (49). 

We showed that IL-12 primes the mice through establishment of Th1 immune response against *L. major* parasites. Comparison of the outcomes in the pleish-dom/pIL-12 and pleish-dom groups validated the critical role of IL-12 in a shift of mixed Th1/Th2 cells to predominant Th1 cells, which produce a high level of IFN-γ. Infected cells in the presence of IFN-γ are stimulated to make TNF-α, leading to intracellular killing of parasites (50). Spleen parasite burden was markedly reduced in the presence of IL-12 in the pleish-dom/pIL-12 group. Indeed, the presence of IL-12 and elevated levels of IFN-γ has been able to be a preventive factor to visceralize *L. major*.

It has been revealed that the IL-10 with immunosuppressive activity on the macrophages contributes to progression of disease in cutaneous leishmaniasis. This inhibitory effect leads to inability of macrophages to eliminate intracellular amastigotes (51). 

Mice vaccinated with pleish-dom/pIL-12 developed localized and controlled lesions, whereas severe lesions were observed in control group mice leading to the progressive forms of the disease including infection that is not observed in the vaccinated group.

Histopathology examination did not show any visceralized infection in the vaccinated group. Progressive disease in control groups led to 83% decease (5 from 6 mice) of remaining mice up to 3 months post-infection. But all mice of the pleish-dom/pIL-12 group survived during this time.

The results indicated that there is an association between the type of immune response and the parasite burden in the spleen. Induction of Th1 immune response resulted in inhibition of parasite development in the spleen. Since the IFN-γ/IL-10 ratio enumerates as an indicator to determine the type of immune response, this ratio was calculated and compared for different groups of study. The mice vaccinated with pleish-dom plus pIL-12 mounted an IFN-γ -dominated Th1 response with the highest IFN-γ/IL-10 ratio, which is in agreement with the splenic load of parasites versus the low IFN-γ/IL-10 ratio with the high numbers of parasites in the spleen for control groups. The mice of pleish-dom/pIL-12 groups showed an approximately 700-fold reduction in parasite burden in the spleen. The results suggested that although the elevated IFN-γ has enabled the mice vaccinated with pleish-dom to form granulomas in the liver, it did not lead to significant decrease of parasite burden in the spleen in comparison with the control groups. It showed that IFN-γ alone might be insufficient for induction of the leishmanicidal activity.

The cellular immune response is considered as the main defense mechanism against *L. major* infection (52). Histopathological examination at the site of the infection in different groups of mice was performed for evaluation of cellular immune response. The results confirmed that morphological features in the lesions correlate with induced immune response phenotype. Granulomas were formed as a result of a Th1 immune response in vaccinated mice. 

## Conclusion

The current study indicated that the designed DNA vaccine, which expresses pooled predominant antigens, was able to induce an immune response against *L. major* that could be boosted significantly by IL-12. These findings may help to design a more efficient pattern in DNA vaccine-based therapy.
